# Organ-specific inhibition of metastatic colon carcinoma by CXCR3 antagonism

**DOI:** 10.1038/sj.bjc.6605078

**Published:** 2009-05-12

**Authors:** B Cambien, B F Karimdjee, P Richard-Fiardo, H Bziouech, R Barthel, M A Millet, V Martini, D Birnbaum, J Y Scoazec, J Abello, T Al Saati, M G Johnson, T J Sullivan, J C Medina, T L Collins, A Schmid-Alliana, H Schmid-Antomarchi

**Affiliations:** 1 Université de Nice Sophia Antipolis, UFR Sciences, Nice F-06002, France; 2 INSERM U576, Nice F-06202, France; 3 CHU, Hôpital de l'Archet II, Service de Chirurgie Générale et Cancérologie Digestive, Nice F-06202, France; 4 INSERM UMR599, Marseille F-13009, France; 5 INSERM U865, Lyon F-69372, France; 6 Plateau Technique d'Histopathologie Expérimentale de l'IFR30, Toulouse F-31300, France; 7 AMGEN, South San Francisco, CA94080, USA

**Keywords:** chemokine receptor, metastasis, colon cancer, anti-tumour strategy, animal model

## Abstract

Liver and lung metastases are the predominant cause of colorectal cancer (CRC)-related mortality. Recent research has indicated that CXCR3/chemokines interactions that orchestrate haematopoetic cell movement are implicated in the metastatic process of malignant tumours, including that of CRC cells to lymph nodes. To date, however, the contribution of CXCR3 to liver and lung metastasis in CRC has not been addressed. To determine whether CXCR3 receptors regulate malignancy-related properties of CRC cells, we have used CXCR3-expressing CRC cell lines of human (HT29 cells) and murine (C26 cells) origins that enable the development of liver and lung metastases when injected into immunodeficient and immunocompetent mice, respectively, and assessed the effect of CXCR3 blockade using AMG487, a small molecular weight antagonist. *In vitro*, activation of CXCR3 on human and mouse CRC cells by its cognate ligands induced migratory and growth responses, both activities being abrogated by AMG487. *In vivo*, systemic CXCR3 antagonism by preventive or curative treatments with AMG487 markedly inhibited the implantation and the growth of human and mouse CRC cells within lung without affecting that in the liver. In addition, we measured increased levels of CXCR3 and ligands expression within lung nodules compared with liver tumours. Altogether, our findings indicate that activation of CXCR3 receptors by its cognate ligands facilitates the implantation and the progression of CRC cells within lung tissues and that inhibition of this axis decreases pulmonary metastasis of CRC in two murine tumour models.

Similar to the majority of solid malignant tumours, colorectal carcinoma (CRC) develops metastases that are the major source of morbidity and mortality. More than one-half of all patients develop metastases, especially in the liver (50% of patients) and lung (15% of patients) (Chambers *et al*, 2002). Owing to the inability to eradicate the metastases with the currently available therapies, new strategies are needed to prevent and eradicate metastases in the target organs and to improve the survival rate of patients. There is mounting evidence that the organ-specific metastasis is governed, in part, by interactions between chemokine receptors expressed on cancer cells and the corresponding chemokines secreted within the target organs (Balkwill, 2004). Of particular interest, the CXCR4/CXCL12 interaction was the first ligand–receptor pair identified to play a critical role in determining the metastatic destination of breast cancer cells to the lungs (Müller *et al*, 2001) and has, since then, been involved in the metastatic spread of many other cancer cells to various target organs (Payne and Cornelius, 2002; Taichman *et al*, 2002; Smith *et al*, 2004; Perissinotto *et al*, 2005; Schimanski *et al*, 2005; Sun *et al*, 2005). Beside the effect on tumour dissemination, CXCR4 as well as other chemokine receptors have also been implicated in various aspects of tumour progression such as cancer cell proliferation and survival as well as in the regulation of the leukocytic and endothelial milieu within the tumour environment (Vlahakis *et al*, 2002; Koizumi *et al*, 2007).

Among the other chemokine receptors under study in this context, CXCR3 has been identified in various cancer cells (Jones *et al*, 2000; Goldberg-Bittman *et al*, 2004; Ben-Baruch, 2006; Engl *et al*, 2006; Albini and Sporn, 2007) and was recently reported to play an important role in breast cancer metastasis to the lung (Müller *et al*, 2001; Kawada *et al*, 2004) as well as in melanoma and colon cancer metastasis to the lymph nodes (Walser *et al*, 2006; Kawada *et al*, 2007). However, so far, the relevance of CXCR3 expression levels in colon cancer metastasis to liver and lung, which remains the major leading cause of death in this malignancy, has not been investigated.

This study aimed precisely at getting new insights into the role played by CXCR3/ligands interactions in the metastatic development of CRC cells within both target organs. *In vitro*, we have analysed how CXCR3 activation by its cognate ligands on CRC cells of human and mouse origins could lead to malignancy-related activities, namely proliferation, survival and migration. Besides, to take into account the heterogeneity of target organs, microenvironmental factors and immune mechanisms, we have developed models of lung and liver metastatic colon cancer using human and mouse tumour cells into immunodeficient and immunocompetent mice. Using a pharmacologic CXCR3 antagonist, we have assessed the contribution of CXCR3 receptors to the formation and the growth of liver and lung metastases and evaluated the therapeutic potential of systemic CXCR3 blockade as an anti-metastatic strategy for this malignancy.

## Materials and methods

### Tumour cell lines and experimental animals

Human HT29 and mouse C26 colon carcinoma cells were maintained in McCoy's 5A medium and murine B16F10 melanoma in MEM medium, both supplemented with 10% heat-inactivated foetal bovine serum (FBS). Female SCID, Nude and BALB/c mice, 6–8-weeks old, were purchased from Harlan (Gannat, France). All of the procedures involving laboratory animals and their care were conducted in accordance with institutional guidelines under veterinary supervision.

### Western blot analysis

Colorectal carcinoma and B16F10 cells were grown in serum-containing medium for 96 h. Cell lysates were subjected to SDS–PAGE and western blotting with rabbit polyclonal antibodies against CXCR3 (Santa Cruz Biotechnology, Tebu, Le Perray-en-Yvelines, France). Detection was done by secondary HRP-conjugated goat antibodies against rabbit IgG (Dako, Trappes, France). The blot was reprobed with anti-Hsp-60 antibodies (Santa Cruz Biotechnology, Santa Cruz, CA, USA) to control for equal loading. Bands were visualised by chemiluminescence-enhanced reaction (Amersham, Les Ulis, France).

### TaqMan real-time PCR experiments

Total RNA from human colorectal cancer and healthy colon, from healthy mouse and metastatic tissues as well as colon carcinoma cell lines was extracted using RNeasy kit (Qiagen, Courtaboeuf, France) and transcribed into cDNA using the Superscript III enzyme (Invitrogen, Cergy Pontoise, France). Real-time PCR was performed in an ABI PRISM 7000 and carried out using TaqMan gene expression assays (Applied Biosystem, Courtaboeuf, France). Cycle parameters were 50°C for 2 min, 95°C for 10 min followed by 40 cycles of 95°C for 15 s and 60°C for 1 min. Relative levels in mRNA expression were determined using Δ*C*_T_ values obtained by subtracting *C*_T_ control (human actin, mouse rasIP) from *C*_T_ target gene (human and mouse CXCR3, CXCL9, CXCL10 or CXCL11), measured in the same RNA preparation.

### *In vitro* chemotaxis assay

Chemotactic responses of colon cancer cells were evaluated by using 24-well cell migration chambers with 8*μ*m pore inserts (Becton Dickinson, San Jose, CA, USA) coated with 6.5 *μ*g ml^−1^ fibronectin (Sigma, St Louis, MO, USA) or with 50 *μ*g ml^−1^ collagen (Becton Dickinson) for the C26 cells or the HT29 cells, respectively. Colon cancer cells treated or not with 1 *μ*mol l^−1^ AMG487 or dimethyl sulfoxide (DMSO) vehicle were placed in the upper well (5 × 10^4^ cells) and various concentrations of human or mouse rCXCL9, rCXCL10 and rCXCL11 (all from R&D systems) were added to the lower wells. After incubation of the plates for 18 h (C26 cells) or for 40 h (HT29 cells) at 37°C in 5% CO_2_ atmosphere, non-migrated cells were removed from the upper well and the migrated cells collected on the lower side of the insert were stained using crystal violet dye and enumerated. Migration index was calculated as the ratio of the number of migrated cells in chemoattractant-containing wells divided by the number of cells that migrated to base medium alone.

### *In vitro* proliferation assay

Colon cancer cells were seeded at a density of 10^4^ cells cm^−2^ and incubated either in serum-enriched medium or in base medium (containing 0.1% bovine serum albumin, BSA) supplemented or not with various concentrations of rCXCL9, rCXCL10 and rCXCL11 (all from R&D systems) for the indicated periods of time before being either trypsin-detached, collected and enumerated or re-fed with fresh medium for 3 days, harvested and enumerated. The morphology of the CRC cells was observed through an inverted optical microscope (Leica, Wetzlar, Germany) at × 20 magnification, and photographs were taken at day 7.

### Caspase activity

CRC cells were grown as indicated in the proliferation assay. A positive control of apoptosis was included by treating the cells for 6 h with 1 *μ*M staurosporine in serum-enriched medium. Cells were then lysed and sonicated in 50 mM Hepes pH 7.5, 150 mM NaCl, 20 mM EDTA, 1 mM PMSF, 10 *μ*g ml^−1^ leupeptin, 2 *μ*g ml^−1^ aprotinin and 0.2% NP-40. Equal amount of lysates were mixed in substrate buffer (50 mM Hepes, 100 mM NaCl, 1 mM EDTA, 10% sucrose, 0.5% CHAPS, 5 mM dithiothreitol) with Ac-DEVD-AMC substrate (ALEXIS Biochemicals, Illkirch, France) and caspase-3/7 substrate in a microtiter plate. Production of fluorigenic substrate was measured continuously at 37°C in a spectrophotometer Ascent Fluoroskan (Thermo Fischer Scientific, Cergy Pontoise, France) and the caspase activity (expressed as U mg^−1^ of protein) was defined as the amount of enzyme cleaving 1 nmol of substrate/min.

### AMG487 formulation

AMG487 was kindly provided by Amgen (South San Francisco, CA, USA). The *in vitro* formulation of AMG487 was prepared as a 10 mmol l^−1^ stock with DMSO and the antagonist was used at the final concentration of 1 *μ*mol l^−1^ for the indicated periods of time in the *in vitro* assays. For the preventive treatment with AMG487, tumour cells growing in culture were washed and re-fed with fresh growth medium containing 1 *μ*mol l^−1^ AMG487 or DMSO vehicle. After 18 h at 37°C, the tumour cells were washed and processed as usual for injection into mice. The *in vivo* formulation of AMG487 was prepared in 20% of hydroxypropyl-*β*-cyclodextrin (Sigma) as described earlier (Walser *et al*, 2006; Wijtmans *et al*, 2008) and used to s.c. treat mice twice daily at 5 mg kg^−1^.

### Mouse models of pulmonary and liver metastasis

For the induction of pulmonary metastases, HT29 cells (2 × 10^6^) or C26 cells (3 × 10^4^) were delivered by intravenous tail injection into SCID or Balb/c mice, respectively (Vitale *et al*, 2007). For the polymetastatic liver model, 2 × 10^6^ HT29 cells or 5 × 10^4^ C26 cells were injected intravenously into the portal vein of Nude or Balb/c mice, respectively. The curative treatment of the mice-bearing metastases was achieved by treating the animals twice daily on days +5 to +12 (syngeneic C26 model) or on days +5 to +23 (immunodeficient HT29 model) with subcutaneous injections of 5 mg kg^−1^ of AMG487 or vehicle. The preventive treatment to antagonise CXCR3 was performed by incubating the colon cancer cells with 1 *μ*mol l^−1^ of AMG487 or vehicle for 18 h *in vitro* before their injection into mice and by treating concomitantly the animals twice daily on days −1 and 0 with subcutaneous injections of 5 mg kg^−1^ of AMG487 or vehicle. At sacrifice, complete post-mortem examinations were performed by a veterinary pathologist. Lungs were inflated and injected through tracheal cannulation either with 10% neutral-buffered formalin before being processed for routine histology or with 7% India Ink dye that reveals white tumour nodules against a black lung background. The extent of tumour development in the lungs was assessed by recording the number and measuring the tumour nodules visible on the pleural surface. In the hepatic model, total liver weight was recorded and compared between the treated and the control groups of mice before formalin-fixation for morphologic evaluation and immunohistochemistry.

### Histology/immunohistochemistry

Formalin-fixed, paraffin-embedded sections of colon cancer metastatic tissues were stained with haematoxylin/eosin and alcian blue for morphologic evaluation. Immunostaining of CXCR3 was performed with anti-human CXCR3 mAb (BD Pharmingen, Le Pont de Claix, France) by the avidin–biotin complex immunoperoxidase method following microwave antigen retrieval. The primary antibody was replaced with phosphate-buffered saline in adjacent tissue sections as negative control. Expression of CXCR3 in human tonsil was used as positive control.

### Statistical analysis

Results are expressed as mean±s.e.m. and analysed using the unpaired Student's *t*-test or ANOVA test with Tukey–Kramer post test.

## Results

### CXCR3 expression in colorectal carcinoma

**Figure 1:**
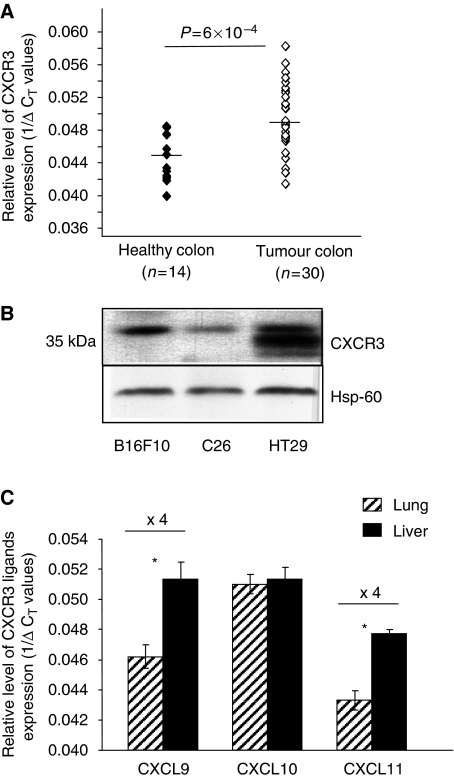
Expression of CXCR3 in human and mouse colon carcinoma. (**A**) Quantitative RT–PCR analysis of CXCR3 in surgical resection pieces of human colon carcinoma (*n*=30) compared with healthy colon tissues (*n*=14). The relative levels of CXCR3 expression were calculated using standard curves and expressed as 1/Δ*C*_T_. Δ*C*_T_ values were calculated by subtracting *C*_T_ of normalising gene from *C*_T_ of target gene, measured in the same RNA preparation. (**B**) Western blot analysis of CXCR3 expression in murine C26 and human HT29 colon carcinoma cells, melanoma B16F10 cells being used as a positive control. (**C**) Expression of CXCR3 ligands in mouse healthy lung (hatched bars) and healthy liver (black bars). Relative levels of expression are determined by quantitative RT–PCR analysis using mouse rasIp as normalising gene. (*n*=6 mice/group). ^*^P<0.01, ^**^P<0.001.

To determine whether CXCR3 is expressed in colon carcinoma (CRC), we analysed by quantitative RT–PCR (real-time quantitative reverse transcription polymerase chain reaction) the expression levels of CXCR3 on surgical re-section pieces of human colon carcinoma and on corresponding healthy colon tissues. Our analysis indicated that the CXCR3 receptor is expressed in healthy human colon tissues (*n*=14) and moderately over-expressed in the biopsies of CRC (*n*=30) (Figure 1A). To address the question whether CXCR3 receptors regulate malignancy-related properties of CRC cells such as invasion and metastasis formation, we have extended our analyses to CRC cell lines of human (HT29 cells) and murine (C26 cells) origins that enable the development of liver and lung metastases when injected into immunodeficient and immunocompetent mice, respectively. Protein expression of CXCR3 was detected by western blotting in human HT29 cells as well as in murine C26 cells (Figure 1B); B16F10 melanoma cells providing a positive control of CXCR3 protein expression as described earlier (Kawada *et al*, 2004). We next evaluated the ability of the mouse healthy lung and liver to produce the corresponding CXCR3 ligands. Analyses by quantitative RT–PCR done on mouse CXCL9, CXCL10 and CXCL11 indicated that all three chemokines were expressed by healthy lung and liver tissues (Figure 1C).

### CXCR3 mediates the migration of colorectal carcinoma cells induced by the CXCR3 ligands

**Figure 2:**
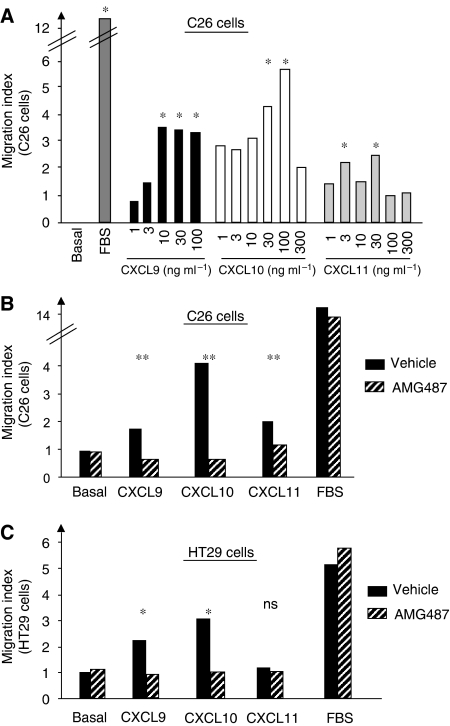
Chemokine-induced migration of colon carcinoma cells. C26 cells (**A** and **B**) or HT29 cells (**C**) were placed in the upper well of a migration chamber and assayed for chemotaxis in response to the indicated concentrations of recombinant CXCL9, CXCL10, CXCL11, to base medium alone (basal) or to serum-enriched medium (FBS). (**B**) Effect of CXCR3 antagonism by AMG487 on the chemotactic response of C26 cells to CXCL9 (10 ng ml^−1^), to CXCL10 (30 ng ml^−1^) or to CXCL11 (30 ng ml^−1^). (**C**) Impact of CXCR3 blockade on the migratory response of HT29 cells to CXCL9 (10 ng ml^−1^), to CXCL10 (20 ng ml^−1^) or to CXCL11 (10 ng ml^−1^). Results represent the mean±s.e.m. of six determinations. ^*^P<0.05, ^**^P<0.001.

The expression of CXCR3 receptors by human and murine colon carcinoma cells has led us to determine the ability of the corresponding ligands (mouse CXCL9, CXCL10 and CXCL11) to induce their migration *in vitro*. To this end, C26 cells as well as HT29 cells were harvested, placed into cell migration chambers and allowed to migrate towards various concentrations of CXCL9, CXCL10, CXCL11 or base medium alone. Figure 2A shows that C26 cells migrated to each of the chemokines in a dose-dependent manner compared with base medium alone. The maximum migratory response was observed in response to CXCL10 (30–100 ng ml^−1^) but also occurred at 10–100 ng ml^−1^ with CXCL9 and at 1–30 ng ml^−1^ with CXCL11. These results suggest that CXCR3 activation by its cognate chemokines can induce invasion-related properties in C26 cells.

Next, we analysed the ability of AMG487 to affect the chemokine-induced migration of the cancer cells by performing the migration assay in the presence of 1 *μ*mol l^−1^ of AMG487 or vehicle and by testing the capacity of the cells to respond to the most effective dose of each chemokine. As shown in Figure 2B, the CXCR3 antagonist significantly inhibited the migratory responses of the C26 cells to each of the three ligands, thus confirming the specificity of the chemotactic response induced by the chemokines.

We next examined whether the human HT29 colon cancer cells exhibited similar abilities as the mouse C26 cells to migrate through their CXCR3 receptors. As for the murine cells, the chemotactic responses towards various concentrations of chemokines were tested and confirmed the migratory ability of the human tumour cells in response to two of the three ligands (CXCL9, CXCL10 but not CXCL11) (Figure 2C). The maximum chemotaxis occurred in response to 10 ng ml^−1^ CXCL10 and to 30 ng ml^−1^ CXCL9. Additionally, in line with our previous results in the C26 cells, the chemokine-induced migration of the HT29 cells was blocked by the molecular CXCR3 antagonist. These data suggest that CXCR3 activation by its cognate ligands appears as a common feature of the CRC cells from distinct origins and that it could mediate the invasion-related properties of colon cancer cells.

### CXCR3 mediates the chemokine-induced survival of the C26 tumour cells

**Figure 3:**
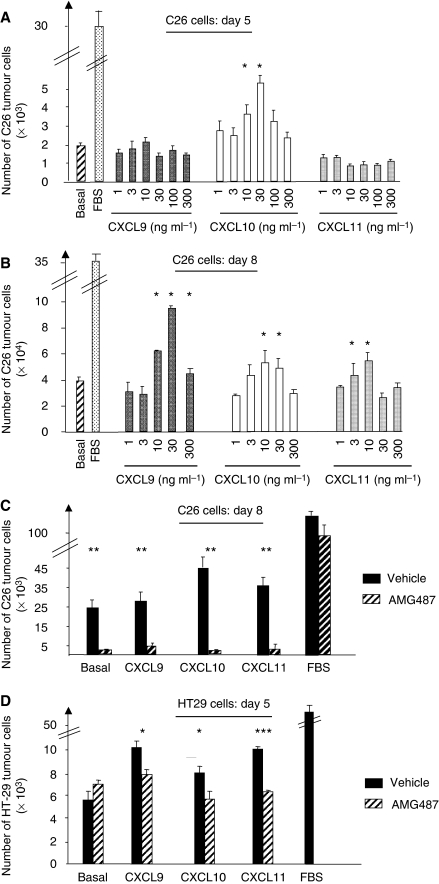
Effect of CXCR3 activation on the proliferation of colon carcinoma cells. (**A**) C26 cells proliferation on serum starvation was assessed in response to a 5-day treatment with the indicated concentrations of either CXCL9 (black bars), or CXCL10 (white bars), or CXCL11 (gray bars), or with base medium alone (hatched bars) or with serum-enriched medium (dotted bars). (**B**) C26 tumour cells were grown for 5 days as described above (panel **A**) before being re-fed fresh medium for 3 days and enumerated. (**C** and **D**) Effect of CXCR3 antagonism by AMG487 on the growth response of C26 cells (**C**) induced by CXCL9 (30 ng ml^−1^), by CXCL10 (10 ng ml^−1^) or by CXCL11 (10 ng ml^−1^) or on the proliferation of HT29 cells (**D**) in response to CXCL9 (3 ng ml^−1^), to CXCL10 (3 ng ml^−1^) or to CXCL11 (30 ng ml^−1^). Results are expressed as mean ± s.e.m. from six independent determinations. ^*^P<0.05, ^**^P<0.001, ^***^P<0.0001.

**Figure 4:**
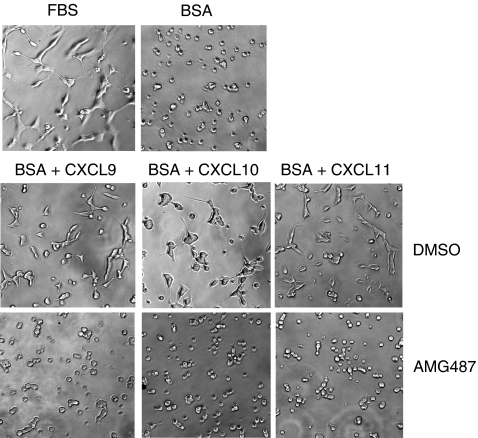
Representative optical photographs of C26 cells morphology in proliferation/survival assays. The C26 cells grown in serum-enriched medium showed complete attachment to the surface (FBS) but underwent detachment followed by cell death on serum starvation (BSA). Supplementation of the serum-deprived medium with either CXCL9, or CXCL10 or CXCL11 allowed the majority of the tumour cells to reverse this non-attached phenotype and to be protected from death. This benefit in survival was abrogated when tumour cells were co-incubated in the presence of AMG487. The morphology of the CRC cells was observed at day 7 through an inverted optical microscope (Leica, Germany) at × 20 magnification. Similar observations were done with the HT29 tumour cell line within 5 days of culture.

To establish whether CXCL9, CXCL10, CXCL11 can modulate the growth/survival ability of the colon cancer cell line in culture, we examined and quantified cell growth after plating C26 and HT29 cells for 5 days at low density in base medium alone (serum starvation) or supplemented with various concentrations of CXCL9, CXCL10 or CXCL11. For murine cells, we observed that only CXCL10 could significantly increase cell survival within 5 days of serum starvation (Figure 3A). In contrast, a significant increase in viability compared with the base medium alone was observed when the cells were first treated with CXCL9, CXCL10 or CXCL11 during the 5-day starvation period before being re-fed with fresh medium for 3 days (Figures 3B and 4). As observed under microscope, the C26 cells grown in serum-enriched medium showed complete attachment to the surface (Figure 4, FBS condition). In contrast, tumour cells that underwent serum starvation for 5 days before being re-fed fresh medium (BSA condition), looked barely attached to the surface and were mostly found in suspension as reflected in the enumerated adherent cells (Figure 3C). Simply supplementing the BSA condition either with CXCL9, CXCL10 or CXCL11 allowed the cells to reverse this non-attached phenotype and to survive in culture. Clearly, however, this gain in proliferation/survival was abrogated by AMG487 treatment, thus indicating the involvement of CXCR3 in the chemokine-induced proliferation of the C26 cells (Figures 3C and 4).

Similar growth ability afforded by chemokine treatments on serum starvation was found for the human HT29 cells as shown in Figure 3D, the benefit being already detectable after a 5-day treatment of the cells. Here again, CXCR3 antagonism through AMG487 abrogated this cell response (Figure 3D). These findings suggest that both human and murine colon cancer cells share the ability to respond to CXCR3 activation in terms of proliferation.

Because serum starvation may represent proapoptotic conditions for the cells as depicted in Figure 4, we next evaluated whether tumour cells could undergo caspase-dependent cell death in base medium (BSA condition) but be protected by chemokine treatments. As a positive control of apoptosis, CRC cells were subjected to a proapoptotic treatment with staurosporine and rapidly underwent apoptosis as revealed by measurement of caspases-3/7 activation (data not shown). However, we failed to detect any change in caspase activation when CRC cells were subjected to serum deprivation. These data suggest that serum-starvation-triggered cell death is associated with a caspase-independent mechanism in HT29 and C26 colon cancer cells but that a protection from death is afforded by CXCR3 activation in these cells.

### Systemic CXCR3 antagonism reduces pre-established lung metastases but not liver metastases from colon cancer

**Figure 5:**
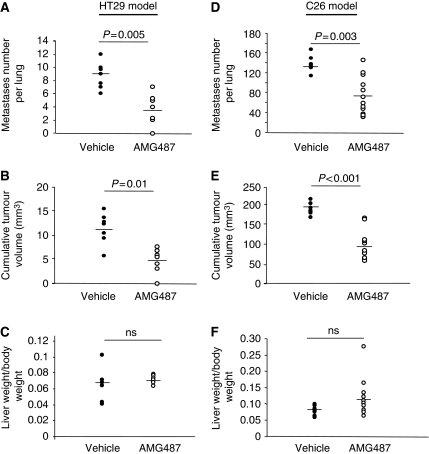
Curative effect of AMG487 on the development of pre-established colon cancer metastases. Mice were injected with HT29 cells (**A**–**C**) or with C26 cells (**D**–**F**) either into the tail vein (**A**, **B**, **D** and **E**) or into the portal vein (**C** and **F**) before receiving subcutaneous injections of AMG487 or vehicle as described in ‘Materials and Methods’ section. On sacrifice, the extent of tumour development was assessed by recording the number of pulmonary metastases (**A** and **D**), by measuring the cumulative tumour volume in the lung (**B** and **E**) and by weighing the livers in the hepatic models (**C** and **F**). The Student's *t*-test was used for statistical analysis: ns, not significant. (*n*=7–12 mice per group).

On the basis of inhibitory effect of AMG487 on the proliferative ability of both HT29 and C26 cells in culture, we seeked to evaluate the potential-blocking effect of this coumpound on pre-established lung and liver metastases in immunodeficient and immunocompetent mice. At day 0, the CXCR3-positive HT29 cells or C26 cells were injected either into the tail vein or into the portal vein of mice to generate lung or liver metastases, respectively. Mice were then treated twice daily on days +5 to +23 (HT29 model) or on days +5 to +12 (C26 model) with s.c. injections of 5 mg kg^−1^ of AMG487 or vehicle, dose described earlier to be therapeutic (Walser *et al*, 2006). At sacrifice, the extent of tumour development in the lung was assessed by measuring and recording the number of tumour nodules visible on the pleural surface. All the mice from both the vehicle- and the AMG487-treated groups developed pulmonary metastases (Figure 5A and D). However, the AMG487-treated mice exhibited fewer pulmonary nodules than the control mice in both the models: a 62% reduction in the human model (3.3 *vs* 8.8, *P*=0.005) and 42% less nodules in the mouse model (78 *vs* 134, *P*=0.003). The inhibitory effect of AMG487 treatment was also detectable in the cumulative tumour volume with a reduction by 58% in the lungs of the HT29-injected immunodeficient mice (4.6 *vs* 10.9 mm^3^, *P*=0.01) (Figure 5B) and by 51% (93 *vs* 189 mm^3^, *P*<0.001) in the lungs of the C26-injected syngeneic mice (Figure 5E). These data indicate that CXCR3 is involved in the metastatic development of CRC cells within lung tissues and that blockade of the CXCR3/ligands axis significantly reduces the progression of pre-established pulmonary nodules. Given that liver is a major target organ in the CRC malignancy, we next assessed the anti-tumour potential of AMG487 on liver metastases-bearing mice. Strikingly, however, systemic antagonism performed with AMG487, at the dose shown earlier to be effective in the lungs, did not lead to any significant decrease in the tumour development in the liver, both in the HT29 and in the C26 tumour models (Figure 5C and F), thus suggesting that the metastatic progression of CRC cells within the liver might rely on distinct mechanisms than in the lungs.

### CXCR3 antagonism affects the metastatic spread of C26 cells to the lungs but not to the liver

**Figure 6:**
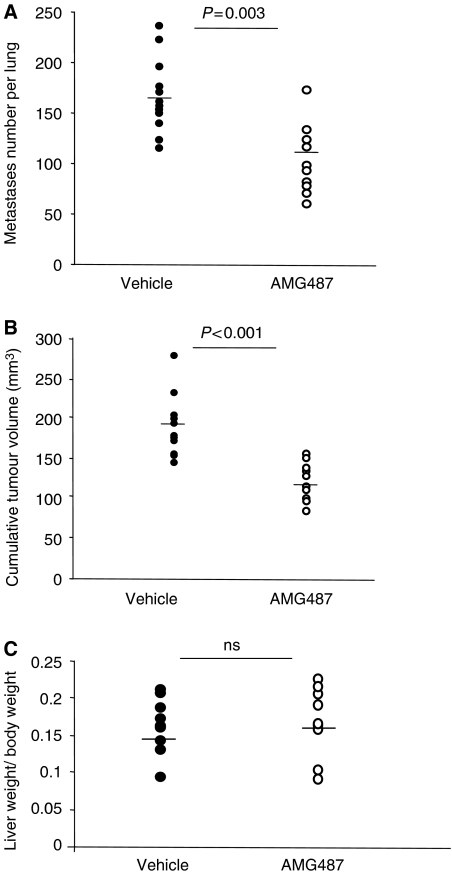
Inhibition of colon carcinoma metastases by preventive treatment with the antagonist AMG487. The preventive treatment to antagonise CXCR3 consisted in treating with AMG487 or vehicle both the C26 cells *in vitro* before their inoculation and the mice on days −1 and 0 as described in ‘Materials and Methods’ section. At day 0, C26 cells were then injected into the tail vein to generate pulmonary metastases (**A** and **B**) or into the portal vein for hepatic metastases (**C**). Twelve days later, the extent of tumour development was assessed by recording the number of tumour nodules visible in the lungs (**A**), by calculating the cumulative tumour volume (**B**) and by weighing the livers (**C**). The Student's *t*-test was used for statistical analysis: ns, not significant. (*n*=8–12 mice per group).

On the basis of chemotactic-promoting effect of CXCR3/ligands interactions on CRC cells, we next investigated the contribution of this axis in the implantation of colon carcinoma within lungs and liver by developing a preventive blockade of CXCR3 before tumour cell inoculation into mice. As CXCR3 antagonism may not only impact on tumour cells but also on CXCR3-dependent immune mechanisms as well, working with the immunocompetent metastatic models appeared more appropriate to assess the anti-metastatic potential of this strategy. Therefore, the CXCR3-positive C26 colon carcinoma cells were pre-treated with 1 *μ*mol l^−1^ of AMG487 or vehicle for 18 h *in vitro* before being injected into syngeneic BALB/c mice to generate either pulmonary or liver metastases. To reinforce the blockade of CXCR3 at the time of CRC cell implantation within the target organs, mice were also treated twice daily before tumour cell inoculation (on days −1 and 0) with s.c. injections of 5 mg kg^−1^ of AMG487 or vehicle. Twelve days after the inoculation of the C26 tumour cells, the extent of tumour development in both target organs was assessed as described above. As shown in Figure 6, 100% of the mice from both groups developed numerous macroscopic pulmonary foci. However, there was a significant reduction in the overall tumour development in the lungs of the AMG487-treated mice compared with the control group as reflected in both the number of foci (37% reduction; 102 *vs* 164, *P*=0.003) and in the cumulative tumour volume (39% reduction 112 *vs* 185 mm^3^, *P*=0.0002) (Figure 6A and B).

We also evaluated the effect of AMG487 on the implantation of hepatic metastases by C26 cells. As in the lung metastasis model, 100% of the mice from both groups developed numerous macroscopic liver nodules (Figure 6C). In contrast, however, there was no significant difference in the overall tumour load in the livers between both groups, as assessed by liver weight (3.03 *vs* 2.89 g, *P*=0.7).

### Comparative analysis of CXCR3/ ligands expression between liver and lung

**Figure 7:**
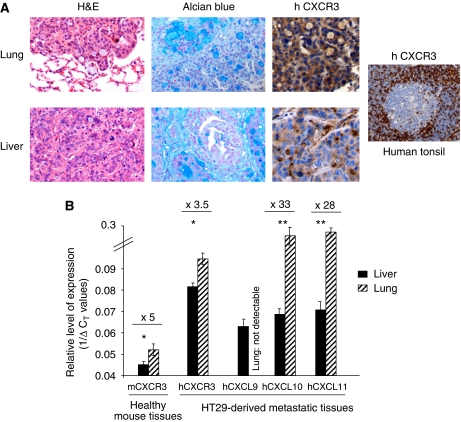
CXCR3 expression within lung and liver metastases of colon carcinoma. (**A**) Histologic analysis of lung and liver metastases. Two months post injection of the HT29 tumour cells, complete post-mortem examinations were performed and tissue samples were fixed in 10% formalin for histology. Sections from lung and liver nodules stained with haematoxylin–eosin are shown (left panels). Alcian blue staining indicates mucin produced by tumour cells (middle panels). Note the difference in human CXCR3 staining (intensity and percent positive cells) between tumour cells in the lung and liver (right panels); positive control for hCXCR3 is shown (human tonsil). Original magnification × 200. (**B**) Quantitative RT–PCR analysis of CXCR3/ligands expression in mouse metastatic liver (black bars) and lung (hatched bars). The relative level of expression of genes is calculated using human actin and mouse rasIp as normalising genes and expressed as 1/Ä*C*_T_ values. ^*^P<0.01, ^**^P<0.001.

The organ-specific inhibition of metastatic colon cancer carcinoma by CXCR3 antagonism has led us to examine CXCR3 expression within lung and liver experimental metastases. To differentiate between CXCR3 expression by the tumour tissues and that by the surrounding healthy organ, we have examined the human HT29 metastases implanted within mouse tissues using antibodies specifically directed against the human protein through immunohistochemistry. As shown in Figure 7A, we observed a marked difference in the human CXCR3 staining between sections of lung and liver metastases both in the percent of CXCR3-positive colon cancer cells and in the expression intensity within the CRC cells cytoplasm. These data indicating a stronger expression of CXCR3 in lung metastases compared with liver foci was further confirmed by quantitative RT–PCR (Figure 7B). Using human probes, we found that lung metastases exhibited a 3.5 increase in the level of human CXCR3 mRNA expression compared with liver nodules. Besides tumour cells, mouse lung tissues also expressed higher mRNA expression levels for the mouse receptor. Strikingly, however, more marked differences were measured in the amount of messengers encoding CXCR3 ligands between both tumours. Except for human CXCL9, in which mRNA levels in the lung tumours were not detectable, we observed significantly higher expressions of human CXCL10 (33-fold increase) and CXCL11 (28-fold increase) by the lung CRC metastases, which suggest a possible mechanism for the lung CRC metastases to promote their development.

## Discussion

Preventing and eradicating metastases in the target organs implies to better understand the mechanisms of organ-specific metastasis. Recently, chemokines receptors have emerged as important mediators in determining the metastatic potential and site-specific spread of cancer cells, their cognate ligands being expressed by the target organs. In colon carcinoma, we have observed that CXCR3 receptors are moderately over-expressed in biopsies of CRC patients compared with healthy colon, confirming the recent report of Kawada *et al* (2007). Although this group recently reported the crucial role played by CXCR3 in the metastatic process of CRC to lymph nodes, the relevance of CXCR3 expression in colon cancer metastasis to liver and lung, which remain the major cause of mortality in this pathology, has not been addressed. To this aim, we have extended our analyses to CRC cell lines of human (HT29 cells) and murine (C26 cells) origins that both express CXCR3 and enable the development of experimental liver and lung metastases when injected into mice. Additionally, we have verified through quantitative RT–PCR the ability of mouse healthy lung and liver to produce the corresponding CXCR3 ligands: CXCL9, CXCL10 and CXCL11. To reach an understanding of the role played by CXCR3 in colon cancer metastasis to liver and lung, we first analysed the direct function of CXCR3 on the tumour cells *in vitro* before assessing its involvement in the implantation and the progession of colon carcinoma within the two main target organs *in vivo*.

The expression of CXCR3 receptors by CRC human tissues and by the human HT29 cells and the murine C26 tumour cells has led us to determine the ability of the corresponding ligands, CXCL9, CXCL10 and CXCL11 to affect the growth and chemotactic responses of HT29 and C26 cells *in vitro*. Here, we show that activation of CXCR3 by its ligands results in both human and mouse tumour cell proliferation, survival and chemotaxis. Taken together, our *in vitro* results directly support the hypothesis that all three CXCR3 ligands display metastasis-promoting activities in CRC cells. Moreover, both motility and growth responses of the colon cancer cells are considerably reduced by AMG487, a small molecular antagonist of CXCR3, thus indicating that CXCR3 may play a major role in the spread and progression of colon cancer metastases *in vivo*.

As a next step, we investigated the relevance of CXCR3 expression levels for colon cancer metastasis to the two main target organs, liver and lung, using two metastatic mouse models. Given that the pharmacologic antagonist of CXCR3 affects the proliferation and the migratory behaviour of both human and murine CRC cell lines *in vitro*, we tested whether a systemic treatment with AMG487 could affect local growth of pre-established metastases. Interestingly, the curative systemic antagonism of CXCR3 markedly reduced both the number of nodules and the cumulative tumour volume within lungs of both HT29 and C26-inoculated mice whereas liver tumours remained unaffected. Thus, CXCR3 appears to contribute to the progression of colon cancer metastases in the lung. In contrast to other published studies that tested the involvement of CXCR3 in cancer progression by performing CXCR3 inhibition at the time of cancer cells implantation within the target organ, our results provide the first evidence of the therapeutic potential of CXCR3-blocking strategies on pre-existing lung metastases. On the basis of the chemotactic effect of CXCR3 ligands on the CRC tumour cells *in vitro*, we further tested the hypothesis that metastases implantation in the liver and in the lung is facilitated by CXCR3 expression on the tumour cell surface. We, therefore, performed experimental metastatic models that selectively address the impact of CXCR3 blockade on the extravasation step of the CRC cells by inoculating the cancer cells into mice through intravenous injections in both the hepatic and the pulmonary models. CRC cells were pre-treated with AMG487 or vehicle before being injected into mice and the preventive action of AMG487 was strengthened by performing a concomitant systemic administration of the antagonist at the time of tumour cells inoculation into mice. Despite the fact that all the treated and control mice exhibited lung metastases at necropsy, we observed a significant reduction in lung metastasis formation in response to the preventive treatment with CXCR3 antagonist. These data indicate that if CXCR3 blockade does not fully prevent CRC cells implantation into the lung, this antagonism is, however, very efficient in limiting this process. However, in contrast to the effects on pulmonary metastasis, the extent of liver metastasis was not affected by the preventive CXCR3 antagonism, pointing to the fact that CXCR3 may mediate organ-specific implantation of colon carcinoma in this model.

Along with our findings, the results by Kawada *et al* (2007) show that targeting CXCR3 is also protective against CRC metastasis to lymph nodes, which underscore the critical role played by this receptor in promoting colon cancer metastasis to various distant organs. Additionally, the organ-specific effect of CXCR3 blockade has also been observed in melanoma (Kawada *et al*, 2004). In that study, CXCR3 was also responsible for lymph nodes metastasis of murine melanoma without affecting that of the lung indicating the role played by CXCR3 in the organ-specific metastasis of distinct cancer cells. Besides colon carcinoma and melanoma, mammary tumour cells were also reported to express CXCR3 that facilitates the development of lung metastasis (Walser *et al*, 2006); therefore, suggesting that CXCR3 may be an important mediator for tumour cells dissemination to the lung.

To gain further insight into the organ-specific inhibition of metastatic CRC through CXCR3 antagonism, we analysed CXCR3 expression within lung and liver tumour nodules. Both our immunohistochemistry and quantitative RT–PCR studies pointed to a higher expression level of the receptor within pulmonary metastases than in the hepatic foci. In addition, higher levels of CXCR3 ligands expression were also measured in lung tumours compared with liver tissues. It is, therefore, tempting to speculate that the CXCR3/chemokines axis may facilitate implantation, growth/survival and expansion of the cancer cells within lung tissues. Apart from the cancer cells, CXCR3 is known to be heterogeneously expressed within tissues (García-López *et al*, 2001). In line with this, we found increased levels of CXCR3 expression within mouse healthy lung tissues compared with healthy liver. It is thus conceivable that CXCR3 blockade through systemic antagonism in the environment of lung metastases contributed to interfere with tumour development. Further elucidating this precise aspect that was beyond the scope of our study, will require approaches performing the specific silencing of CXCR3 on the tumour colon cancer cells.

Despite accumulating evidence of malignancy-related functions of CXCR3 in various cancers, the functions that chemokines and their receptors establish between the tumour cells and their microenvironment are complex, ranging from support to inhibition of the tumourigenesis process (Ben-Baruch, 2006). In particular, the ability of chemokines to attract into tumours leukocytes with potential anti-tumour activities is of importance. Beside its expression on colon cancer cells, CXCR3 is indeed expressed on T lymphocytes, dendritic cells, monocytes, natural killer cells (Qin *et al*, 1998; Katschke *et al*, 2001; Penna *et al*, 2002; Rabin *et al*, 2003) and was reported as an important receptor for the immune response to be optimally executed (Loetscher *et al*, 1996). Likewise, several studies have now clearly showed the anti-tumour activities of the CXCR3 ligands in various syngeneic tumour models (Luster and Leder, 1993; Sgadari *et al*, 1996; Addison *et al*, 2000; Dorsey *et al*, 2002; Feldman *et al*, 2002; Giese *et al*, 2002; Wang *et al*, 2003; Saudemont *et al*, 2005; Walser *et al*, 2007; Zipin-Roitman *et al*, 2007). Consistent with this, we were expecting that neutralising all interactions between CXCR3 and its ligands by systemic CXCR3 antagonism could promote metastasis. However, the extent of tumour development was never significantly enhanced in the AMG487-treated mice compared with the corresponding control group, indicating that the CXCR3 antagonism did not affect the local tumour growth. Although no adverse effect on tumour development was observed in our experiments as well as when AMG487 was systemically administered for 28 days in metastatic breast cancer (Walser *et al*, 2006), further effort will be required to examine this possibility, especially considering that CXCR3 blockade may be useful as a treatment strategy to interfere with metastasis in cancer patients.

In summary, our results show that CXCR3 expression and activation is associated with tumour-promoting activities on colon cancer cells *in vitro*, namely proliferation, survival and migration. The *in vivo* data point to a distinct contribution of the CXCR3/chemokines axis between lung and liver CRC metastases. Preventive systemic treatment with AMG487, a small molecular weight antagonist of CXCR3, significantly reduces metastasis of colon cancer cells to the lung without affecting that to the liver. In addition, our study shows for the first time that using AMG487 as a curative treatment on pre-established metastases markedly reduces tumour development in the lung but not in the liver. Along with the protective action of CXCR3 antagonism on CRC metastasis to lymph nodes reported by Kawada *et al*, our findings provide evidence that targeting the CXCR3/ ligands axis may be beneficial in limiting metastatic colon cancer.
